# Communication in Telehealth: A State-of-the-Art Literature Review of Conversation-Analytic Research

**DOI:** 10.1080/08351813.2024.2305045

**Published:** 2024-04-03

**Authors:** Lucas M. Seuren, Sakari Ilomäki, Evi Dalmaijer, Sara E. Shaw, Wyke J. P. Stommel

**Affiliations:** a Nuffield Department of Primary Care Health Sciences, University of Oxford, UK; b Institute for Better Health, Trillium Health Partners, Canada; c Faculty of Social Sciences, Tampere University, Finland; d Centre for Language Studies, Radboud University, Netherlands

## Abstract

We provide a state-of-the-art review of research on conversation analysis and telehealth. We conducted a systematic review of the literature, focusing on studies that investigate how technology is procedurally consequential for the interaction. We discerned three key topics: the interactional organization, the therapeutic relationship, and the clinical activities of the encounter. The literature on telehealth is highly heterogeneous, with significant differences between text-based care (e.g., via chat or e-mail) and audio(visual) care (e.g., via telephone or video). We discuss the extent to which remote care can be regarded as a demarcated field for study or whether the medium is merely part of the “context,” particularly when investigating hybrid and polymedia forms of care involving multiple technological media.

## Remote healthcare encounters: overview and background

We provide a state-of-the-art review of research on conversation analysis (CA) and *telehealth*. Amending WHO’s definition of *telemedicine*, we define *telehealth* as “the provision of physical, mental, and social healthcare services at a distance with communication (…) conducted between remote healthcare users seeking health services and healthcare providers (client-to-provider telemedicine)” (World Health Organization [WHO], 2021). Telehealth services are typically seen as a form of “mediated interaction,” meaning healthcare provider and patient use a technological medium to establish a joint interactional framework. These media can be (i) text-only, such as e-mail, SMS, or synchronous internet text-messaging, “chat” for short; (ii) audio-only, such as telephone; or (iii) audio-visual, such as videoconferencing. While all three media are distinct, we take them together in this review because (a) healthcare communities (practice, policy, and research) treat telehealth as a coherent unit, i.e., telehealth is a type of members’ category, and (b) the shift from in-person to remote has happened across all media, and there is no a priori reason to prioritize one medium over the other.

In line with Arminen et al. (2016), we take “mediated” as a members’ category, meaning that we focus on telehealth research in which the analysis investigates how the medium is procedurally consequential for the participants (Schegloff, 1991). We thus exclude studies on telehealth encounters that do not analyze them as mediated interaction. For example, Drew (1998, 2006) demonstrated how misalignment occurs in out-of-hours calls based on the expectations of the caller around the clinician’s diagnostic questioning. However, the fact that the encounter is remote and by telephone is, at least with regard to this particular interactional problem, taken for granted.

With the outbreak of the COVID-19 pandemic and the implementation of infection control protocols, healthcare providers were forced to turn to telehealth. This shift came on top of a policy push to move to more remote, hybrid and polymedia care^1^^1^Hybrid is used in health policy and practice to indicate a mix of in-person care and telehealth. Polymedia derives from anthropology to reflect how people use different technological media for specific types of interactional encounters (Madianou & Miller, 2012). in various countries (Australia Digital Health Agency, 2018; BC Ministry of Health, 2015; NHS, 2019; Ontario Ministry of Health, 2019). Some healthcare providers had been keen to make use of remote models to provide more personalized care (convenience for patients being a key motivator; Greenhalgh et al., 2012, 2018). Many patients were happy with remote options, often requesting telephone or text messaging instead of an in-person consultation (Clarke et al., 2022).

Telephone consulting had been routine in primary care and out-of-hours services for decades (Drew, 1998; Heagarty, 1978), and counseling and therapy services had increasingly been using text-based systems (e.g., Ekberg et al., 2013; Stommel, 2012). However, progress in developing video consulting was typically ad hoc, often encountered clinician resistance, involved significant effort in designing and implementing virtual pathways and could be challenging to set up and sustain in the face of limited infrastructure (Greenhalgh et al., 2019). The result was that adoption was slow and resource intensive, with activity limited to a handful of clinical settings. The COVID-19 pandemic changed all that (Ohannessian et al., 2020; Wong et al., 2021). While telephone continued to be the preferred form of telehealth in primary care (Greenhalgh et al., 2022; Hall Dykgraaf et al., 2021), barriers were quickly removed at the outset of the crisis, and novel services such as e-consultations in primary care were quickly scaled up (Chang et al., 2021; Shaw et al., 2021).

The ongoing shift to telehealth provides a unique opportunity for conversation analysts to shape the narrative around healthcare communication. CA has a strong track record of investigating mediated interaction, focused on how participants use different media for social interaction (for recent overviews, see Arminen et al., 2016; Meredith, 2019; Mlynář et al., 2018). The analytical starting point for CA when investigating mediated interaction is that technology does not determine how people act. The question is how the technology is (made) procedurally consequential for the interaction (Arminen et al., 2016). Gibson’s concept of “affordances” (Gibson, 1979) has been used to explain that actors make use of the properties of technology in ways that are relevant and productive for them in achieving their interactional goals (e.g., Hutchby, 2001a, 2001b). These affordances are not objective, definite properties of technology, but are themselves accomplished by participants using them for specific interactional goals (Hutchby, 2001a). The notion of “fractured ecologies” (Luff et al., 2003) also features centrally in CA research on mediated interaction. This captures that participants are not co-present and that their conduct is “fractured from the environment in which it is produced and from the environment in which it is received” (p. 55). The extent of fracturing varies between modalities. Participants have some audio-visual access (albeit delayed) to each other’s environment in video communication, but no audio-visual access in text-based modalities, which can require additional work to maintain the interactional framework (Mlynář et al., 2018).

### The goal of this review

Telehealth offers exciting new ways for CA researchers to understand and support healthcare encounters and for “advancing the field of patient-clinician communication” (van Dael et al., 2022). It requires clinicians and patients to adapt some of their skills and develop new ones. While we cannot assume that telehealth is necessarily different from in-person interaction (Arminen et al., 2016), it is not clear to what extent research on co-present healthcare translates to remote services (Lopriore et al., 2017).

CA research on telehealth has so far not been synthesized. Previous reviews have only addressed telephone calls for emergency lines (Kevoe-Feldman, 2019), helplines (Bloch & Leydon, 2019) or video consultations (Dalley et al., 2020), and all were conducted before the pandemic.

Our goal is therefore two-fold: to synthesize the knowledge, insights, and ideas in CA on telehealth, and to provide a context for future research.

## Methodology

### Literature search

We adapted the protocol designed by Parry and Land (2013) for systematic reviews of conversation-analytic research (see supplementary file A for further detail). We developed a set of search terms and extracted literature from academic databases up to January 10^th^, 2022. After removing duplicates, this resulted in 965 unique publications. Two authors screened the first 200 articles against the following inclusion criteria: (a) used CA as their main or one of their main research methods, (b) focused on naturally occurring interaction in telehealth, either (quasi-)synchronous or asynchronous, (c) analyzed encounters between healthcare professionals and patients, (d) investigated how the remote nature was procedurally consequential for the interaction, (e) used transcripts of their data to support the analysis, (f) involved original, empirical work (e.g., we excluded protocols and reviews) and (g) had been peer-reviewed, including forthcoming, “online first,” publications.

The authors agreed on 96% of cases, with Cohen’s Kappa measure for intercoder reliability at κ = 0.56. The lead author then screened the rest of the collection against established criteria. To complement this systematic search, we conducted a forward citation search and emailed key authors to request additional literature, with nine further articles meeting our inclusion criteria. We updated our search on April 6^th^, 2023, resulting in four additional articles. Our final collection consisted of 41 original articles.

**Figure 1.**
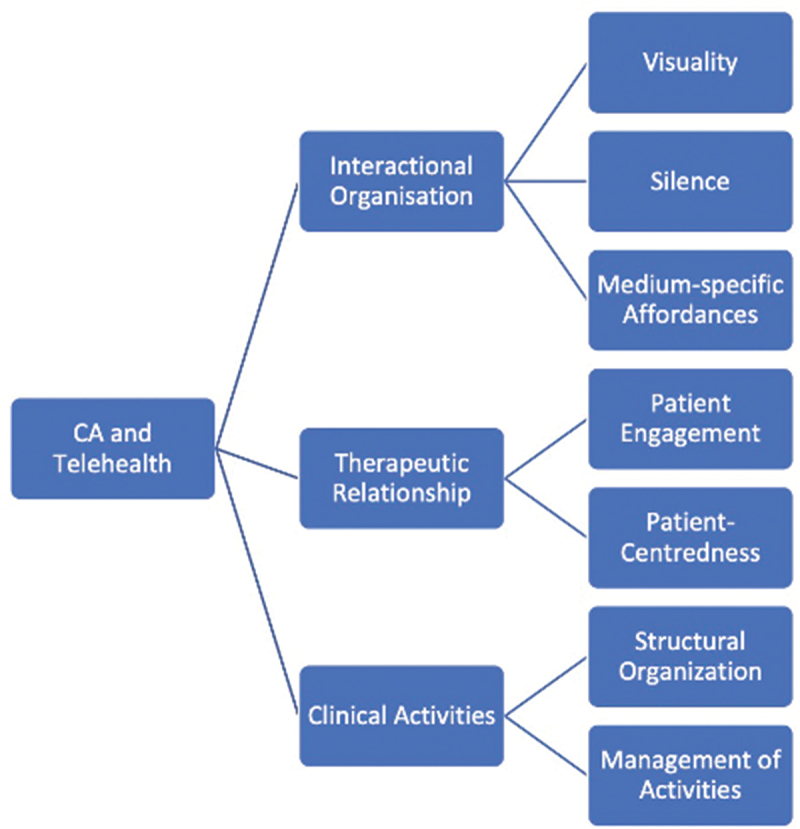
Topical organization of CA and telehealth.

The lead author read all articles, using an open coding system to discern potential commonalities. Codes were discussed in virtual meetings with all coauthors and used to develop analytic topics. This process was iterative, adapting and refining the topics as we returned to the literature and our codes. We settled on three key topics, each with a number of sub-topics, with each publication addressing at least one of these (see Figure 1): how participants manage (i) the interactional organization, (ii) the therapeutic relationship, and (iii) the clinical activities.

Following our discussion of the state-of-the-art, we highlight how CA can inform healthcare policy and practice around telehealth, an area in which CA has much to offer. We reflect on two core issues for this review: (i) our approach to synthesizing telehealth, taking together seemingly disparate media, and (b) the quality and strength of the evidence. In closing, we argue that to have relevance for clinical practice, CA needs to focus on those media that are most used in clinical practice. It also needs to develop tools to investigate hybrid and polymedia forms of care delivery that are becoming increasingly prevalent in healthcare.

## Current state-of-the-art

Telehealth has been studied by CA researchers for nearly 25 years. However, only recently has research on telehealth begun to take off, particularly on video consulting: of the 41 papers we found, 18 were published since 2020 and none before 2005. Of those 18 studies, 16 were on video consulting (see supplementary file B).

CA research on telehealth addresses a range of clinical disciplines (see Table 1). Most studies have been conducted in secondary care and mental health, but within those settings the research is heterogenous, covering, for example, heart failure, cardiology, oncology, diabetes, cognitive behavioral therapy, psychotherapy, and counseling for anxiety or depression. One major limitation of the current body of work is its lack of linguistic and cultural diversity. Most research has been in English and Dutch, accounting for 73% of the total research.

**Table 1.** Summary overview of CA research on telehealth.

Context of Consultation	Number of Articles
Clinical Context	
Primary Care	4
Secondary Care (e.g., cardiology, oncology)	20
Mental Health (e.g., psychotherapy, CBT)	9
General Counseling (e.g., drugs and alcohol)	3
Health Helpline (e.g., NHS Direct/111)	2
Care Homes	3
Telehealth	
Video	22
Telephone	6
Text	12
Chat	8
E-Mail	2
SMS	1
E-Mail & Chat	1
Video & Text	1
Language	
English	17
Dutch	13
Danish	4
Finnish	3
Swedish	1
German	1
Italian	1
Norwegian	1

## Findings

CA research on telehealth generally takes one of two approaches. One group of studies takes commonly known aspects of the technology, such as latency in video calls, and investigates how these shape practices through which participants manage the consultation. The other group uses a data-driven analysis to discern specific practices and activities within the consultation, such as how participants move into closing, and examine how the specificities of the medium affords these practices. In both cases, analysts show that the technology is procedurally consequential for the participants.

We identified three ways in which studies investigate how the affordances of the technology shape the interaction in telehealth. First, studies analyze the interactional organization of remote healthcare, for example, how visuality or the lack thereof affects domains like turn-taking or the participation framework. Second, studies investigate how patients and clinicians build and maintain the therapeutic relationship in remote care, for example, practices used by clinicians to promote engagement. Finally, studies analyze how participants accomplish the various clinical activities in remote encounters, for example, what the lack of a shared physical space means for physical examination. We discuss each of these below.

### Interactional organization of remote healthcare

Our first topic centers on how studies demonstrate that for the participants some aspect of the technology is relevant for how they organize their talk, such as its structural, sequence, or repair organization. Within this, we identified three subtopics: (i) visuality or the lack thereof, (ii) silence or periods of non-talk/texting, and (iii) generic features of specific technologies.

One subtopic that runs through many papers (n = 11) is the affordance of visuality. Studies on video consulting investigate the implications of the visual space for interactional-organizational domains like openings (Due, 2021; Hansen, 2020; Ilomäki & Ruusuvuori, 2020; Nielsen, 2020; Savenstedt et al., 2005; Shaw et al., 2020), closings (Ilomäki & Ruusuvuori, 2020), participation framework (Hansen, 2020; Nielsen, 2020; Pappas & Seale, 2009; Stommel & Stommel, 2021), and turn-taking (Chatwin et al., 2014; Seuren et al., 2021). In studies of telephone consulting, it is precisely the lack of a visual space that is considered consequential (Chatwin et al., 2014).

To initiate a (healthcare) encounter, participants have to establish shared attention. With in-person encounters, clinicians display engagement (or disengagement) through their gaze and body orientation (Heath, 1981; Robinson, 1998). By visually attending to the patient, they make a first step to shared attention or intersubjectivity. Studies have begun to reveal how participants orient to visuality to accomplish this goal over video.

For everyday video interaction, Licoppe (2017) showed how participants initiate greetings at the point where they can see their interlocutor. Similarly, Hansen (2020) and Shaw et al. (2020) found in studies on video consulting that participants provide greetings when they can see their interlocutor, and often withholding greetings until a visual connection is established, even when there is evidence of a working technological connection (i.e., they might be able to hear their co-participant). Doing a greeting thereby provides evidence to the patient that the clinician can see them and vice versa. Ilomäki and Ruusuvuori (2020) and Savenstedt et al. (2005) show that clinicians can provide greetings without seeing the patient, but withhold opening questions until a shared visual space has been established.

Due (2021) offers a similar analysis for situations where clinicians are using telehealth robots, or “RoboDocs,” which they can move in the patient’s physical environment. Clinicians move the robot until they have established a type of F-formation (Due, 2021), in which they can see the patients and any caregivers and thereby establish joint attention (Nielsen, 2020).

While visuality is consequential in different ways in these studies, all demonstrate that participants orient to visuality in how they shape the opening of the consultation. Participants need to be able to see each other and require evidence that they are seen themselves. Greetings can offer such a form of evidence, but participants may seek more explicit confirmation through dedicated sequences (e.g., “can you see me”).

Once closing the interaction, participants do not just produce audible (pre-)closing sequences, but rely on visual techniques: they may wave and visually withdraw (Ilomäki & Ruusuvuori, 2020), or in the case of RoboDoc, drive away, breaking the shared visual space (Due, 2021).

Patients and clinicians also use visuality to establish the participation framework. Other potential participants may be co-present, such as other clinicians (Pappas & Seale, 2009; Shaw et al., 2020), companions with the patient (Nielsen, 2020; Seuren et al., 2024; Shaw et al., 2020; Stommel & Stommel, 2021), or patient and clinician may be together with a remote interpreter (Hansen, 2020). Studies consistently show that whether participants can be seen is consequential for the interaction. Stommel and Stommel (2021) reveal how off-screen participants rarely address remote co-participants and mostly talk to their co-present co-participant. Similarly, Seuren et al. (2024) find that informal carers generally take up a liminal position: while in their data, carers need not be off-screen, they did remain in the background and mostly only performed supportive actions (e.g., handling the technology, doing a physical assessment). Hansen (2020) shows that when interpreters cannot see all participants, they have difficulty in managing who is talking to whom, and thus what is expected of them. The visual space in these studies is treated as demarcating the participation framework: only those in a talking heads configuration (Licoppe & Morel, 2012) are considered active co-participants. When co-present companions or clinicians need to be involved, they are either continuously visible to all participants (Nielsen, 2020; Pappas & Seale, 2009) or they make themselves visible to become active co-participants (Stommel & Stommel, 2021).

The final domain where visuality affects the clinical encounter is turn-taking. Chatwin et al. (2014) argue that in telephone consultations, the lack of a shared visual space partially explains why patient and clinician have problems distinguishing between inter-turn and intra-turn silences. While in video consultations, participants can see each other, latency may similarly give participants the wrong impression about whether co-participants are or are not about to talk (Seuren et al., 2021).

The second subtopic entails studies that investigate how technology plays a role in the emergence and resolution of silences in healthcare encounters, particularly where they are treated by the participants as problematic (e.g., silences where talk should occur; Olbertz-Siitonen, 2015; Ruhleder & Jordan, 2001). In telephone (Chatwin et al., 2014) and video calls (Ilomaki et al., 2021; Nielsen, 2020; Seuren & Shaw, 2022; Seuren et al., 2021; Shaw et al., 2020), silences between turns are more likely to occur. In chat encounters, extended silences are normal while turns are being typed (Stommel & te Molder, 2015a, 2015b).

Chatwin et al. (2014) show that in telephone-delivered cognitive behavioral therapy, where longer silences can be treated as therapeutic and thus unproblematic, participants sometimes misalign in their treatment of a silence, requiring repair strategies.

Participants in video consultations may have to deal with a different technological problem. Latency can cause participants to perceive silence where talk occurs. Patients seemingly have the primary right to continue, with clinicians producing repair strategies that concede the floor, but there is limited evidence on exactly who gets the right to talk when (Nielsen, 2020; Seuren & Shaw, 2022; Seuren et al., 2021; Shaw et al., 2020). While participants generally resolve these problems within a few turns (Seuren et al., 2021), Ilomaki et al. (2021) show that in video-mediated group health counseling, participants may have different perspectives about the type of overlap that occurs and thus who has rights to talk, which may lead to diminished client participation.

For chat encounters, the problem is different. The time between one post and the next is (at least partially) dependent on the time the respondent takes to type the next post or the first “poster” to type another post. Since the time between contiguous posts may be minutes long (unthinkable in talk), they require the participants’ sequentially local interpretation to determine each time again whether a silence is problematic (e.g., by the counselor producing a pre-closing item), a case of someone producing a lengthy post, or even someone engaging in some other activity (Jager & Stommel, 2017; Stommel & te Molder, 2015a). As the management of silences and turn-taking are of clinical importance (e.g., through client participation and preference organization), how technologies shape possibilities for this is of importance for both clinicians and researchers.

The third subtopic is more heterogeneous, but we gloss it as how platform specific affordances are made procedurally consequential. The systems that participants use for telehealth provide new opportunities for interaction. These can be integrated by the nature of the medium (e.g., spoken talk has prosody, text has punctuation (Stommel & van der Houwen, 2013)), but may also be added to specific platforms (e.g., emoji reactions in some video platforms).

The mobility of RoboDoc is a particularly salient affordance that clinicians can use to shape the interaction. It allows them to move through rooms, establish and reestablish an interactional space, display the locus of their attention, and it even makes the remote clinician in some way an embodied co-presence (Due, 2021, 2022).

The medium of chat offers its own affordances. For one, turn-taking is quasi-synchronous: chat consultations involve a real-time, sustained interaction, but turn composition, transmission, and reception do not occur simultaneously (Schönfeldt & Golato, 2003). This allows participants to combine multiple actions into a single message. Ekberg, Shaw et al. (2016) show how in computer-based CBT, therapists produce a closing assessment to the patient’s emotional turn, and in the same move ask a question, setting up new sequential implications, thereby attending to the patient’s displayed emotion, but closing that off for further discussion. Chat also allows participants to use punctuation marks in their turn design. Stommel and van der Houwen (2013) investigated how counselors use formulations in chat and show how question marks are used to design declarative utterances as requests for confirmation.

Finally, Stommel and te Molder (2015b) investigated the sequential implications of pre-screening questions in chat encounters for how counselors design opening questions. Counselors should know about the client’s problem based on these questions, but designing a question that is appropriate for the client’s knowledge rarely leads to a smooth opening (Cipolletta et al. (2018) raise this point for video counseling as well). While this is a known problem in face-to-face healthcare encounters (Heritage & Robinson, 2006), it seems more difficult in chat when counselor and client have not previously met.

These studies highlight how patients and clinicians use affordances of the technology to organize the interaction. Participants manage similar interactional problems as they would with in-person healthcare encounters (e.g., establishing shared attention), but they rely on different practices. Technologies can also provide unique challenges (e.g., latency in video) for which participants develop solutions.

### Therapeutic relationship in remote healthcare

Our second topic captures how patients and clinicians manage the “therapeutic relationship” in telehealth. We identified two interactional dimensions of this relationship: how clinicians promote patient engagement and how they enact patient-centredness.

CA studies have begun to document how clinicians promote patient engagement in telehealth, especially in chat. The reason engagement is a members’ concern is that technologically-mediated interaction affords distractions and disengagement. A quintessential case is offered by Jager and Stommel (2017), who show that in chat-based counseling, when a session is not going smoothly, clients just drop out. Whereas for an in-person session, a patient would need to physically leave a room, in the relatively impersonal and anonymous environment of chat, leaving merely requires a mouse click. As Jager and Stommel show, one consequence for counselors is that they need to address a problematic interaction without alienating the client, for example, they may claim responsibility for a failure to help the clients.

A series of studies by Ekberg and colleagues highlights how in chat-based counseling, clinicians need to do work to promote patient engagement. At the start of an interaction, clinicians have to engage in expectation management to promote engagement with the clinical process (Ekberg, Barnes et al., 2016). Furthermore, throughout the consultation, when patients display emotions through their contributions, clinicians can use specific practices to promote expansion on that emotion or pivot to other activities (Ekberg et al., 2013; Ekberg, Shaw et al., 2016).

While these studies show that clinicians need do work to promote engagement, text also affords engagement. While asynchronous media such as SMS can pose problems compared to in-person consultations, it allows practitioner and patient to stay in touch and maintain a relationship when regular contact is not possible (Buchholz & Kachele, 2015).

The second dimension of the therapeutic relationship we discerned in the reviewed studies is patient-centredness. CA studies have begun to show that telehealth is no less patient-centered and indeed affords new opportunities for patient-centered care.

Both Cipolletta et al. (2018) and White et al. (2022) investigated patient agency in the management of the agenda, in video and telephone consultations respectively. Both found that clinicians set the agenda and provide limited opportunities for patients to participate, and patients have to create their own interactional opportunities. This could be understood as confirming concerns about patient-centredness: clinicians do not provide much room for the patients’ concerns. However, findings confirm that it is not the medium, but the “projects of activities” of the encounter (e.g., establishing medical problems) that provide for a clinician-led consultation (Robinson, 2003)

Related to agency is autonomy, the ability of patients to manage their own care at home. Studies on video consulting indicate that while there are opportunities for patients to be more involved, the fractured ecology can inhibit the ability of clinicians to support autonomy. Seuren et al. (2020) found that some patients display an eagerness to manage their own health and perform their own physical examination. Video allows them to take more control of their illness. They can perform their own examinations or at least are offered an opportunity to manage their illness in their own home with assistance from a carer. They may resist help from remote clinicians and even co-present carers, treating these offers as challenges to their autonomy and competence. Video can “empower” patients by putting the assessment in their hands (Stommel, van Goor et al., 2020). However, Ilomaki and Ruusuvuori (2022) found that in telehomecare consultations, the set-up of video consulting can also inhibit patient autonomy. In their data, older people are asked about their medication and whether they are taking it. Because nurses cannot see the full environment of the client and their surroundings, they have trouble establishing a shared understanding of medication and other “care-relevant artefacts” in the client’s home. This makes it difficult for nurses to support the client’s independent actions. These studies indicate that the affordances of video impact autonomy in different ways. Video affords patient participation in the consultation, but it can be difficult for clinicians to support patients with this.

Finally, text provides a unique affordance for patient-centredness, as revealed by Stommel (2012) who examined salutations and closings in e-mail counseling. She shows that these offer a new opportunity each time to reestablish or amend the relationship with the patient. By using either the patient’s first or last name, as well as more formal closings (e.g., “with kind regards”) or informal closings (e.g., “best”), clinicians enact an informal or formal relationship respectively. However, it also can lead to new errors. Stommel found that counselors make mistakes with address terms as requested by patients, possibly as a result of copy-pasting or because the fractured ecologies can cause them to forget who the client is.

The CA literature around the therapeutic relationship in telehealth is sparse, and mostly focuses on text-based counseling and therapy. However, these studies reveal that while technological mediation can make it harder to coordinate actions, especially regarding emotions and artifacts, patients and practitioners use the affordances of the medium for interactional practices that (re-)enact and maintain their relationship.

### Clinical activities in remote healthcare

Our third topic captures how participants manage the institutional activities that make up a healthcare encounter (Byrne & Long, 1976; Robinson, 2003). Within this, we identified two subtopics: (i) the overall structural organization of telehealth encounters, including whether there are activities that are distinct from in-person care; and (ii) how participants manage these activities (differently).

In our dataset, two studies focus on the overall structural organization of telehealth encounters. Both show that in telephone consultations, clinicians take a triage approach by organizing the consultation around the presentation of a single acute complaint, with no opportunities for presenting additional complaints (Hewitt et al., 2010; Lopriore et al., 2017). The structural organization is thus streamlined toward assessing whether an in-person consultation is needed or if the complaint can be managed remotely. Lopriore et al. (2017) also reveal how nurses in telephone calls perform an additional safety check before moving into the history taking. This check was needed to assess the caller’s vital signs, to determine if they are severely unwell or experiencing a medical emergency. For in-person consulting (and possibly video), nurses could do this visually, preempting the verbal check, showing the implications of the medium (audio only) for the structural organization of the interaction.

The main body of work on clinical activities in telehealth focuses on physical examinations. This is unsurprising given that it is *the* activity for which the lack of touch seems a particular problem. Studies of video and telephone consulting investigate how participants conduct a physical examination despite this limitation. This work hones in on three interactional problems: (i) how to talk about the patient’s body as a clinical object (Heath, 2006; i.e., how it looks or feels) in a way that is understandable for the patient or their carer and relevant for the clinician (Lopriore et al., 2019; Seuren et al., 2024; Seuren et al., 2020); (ii) how to get the patient or their carer to feel the patient’s body in a clinically appropriate way (Lopriore et al., 2019; Pappas & Seale, 2010; Seuren et al., 2020; Stommel, van Goor et al., 2020); and, in the case of video, (iii) how to make the patient’s body visible (Due, 2022; Due & Lange, 2020; Pappas & Seale, 2010; Seuren & Shaw, 2022; Seuren et al., 2024; Seuren et al., 2020; Stommel, Licoppe et al., 2020; Stommel, van Goor et al., 2020).

These studies show that in both telephone and video, participants routinely have to manage the limited clinician expertise of the patient and/or their carers (Lopriore et al., 2019; Seuren et al., 2024; Seuren et al., 2020;), that is, their lack of professional touch and vision (Goodwin, 1994). Even where a healthcare professional is co-present with the patient, discrepancies in expertise between the co-present clinician and remote specialist can require tailored instruction sequences (Pappas & Seale, 2010). Video might be expected to be easier than telephone (or text), because it offers the additional affordance of visuality; however it brings with it the interactional problem of coordinating the patient’s body and the technology (Seuren & Shaw, 2022; Seuren et al., 2024; Seuren et al., 2020; Stommel, van Goor et al., 2020). Patient and caregiver have to consider whether to move the camera or the patient’s body (Due & Lange, 2020), a decision in which they invariably orient to the mobility of the technology as an affordance: a smartphone or tablet can be moved, Robodoc can move around, whereas a desktop camera is in a fixed position.

The lack of a shared physical space in telehealth also requires adaptations when clinicians want to use physical objects (Licoppe et al., 2017). For example, Seuren et al. (2020) show that in heart failure consultations, patients can take measurements (e.g., blood pressure) on the clinician’s behalf, with the clinician providing instructions on how to use and read instruments. Ekberg et al. (2019) investigate speech and language therapy by video, in which therapists use toys to engage and reward the patients. Patients cannot manipulate the toys directly over video, so Ekberg et al. (2019) show how clinicians instead manipulate the objects themselves when patients produce the desired verbal expressions.

To date, other activities that make up a telehealth encounter have received sparse attention. The opening has been studied by a number of researchers for video and telephone (Ilomäki & Ruusuvuori, 2020; Lopriore et al., 2017; Pappas & Seale, 2009; Stommel et al., 2019).^2^^2^Here we focus on the institutional organization of openings, where participants move from preliminaries to the business at hand (Heath, 1981). Other papers also address the opening of remote healthcare encounters, but focus on the interactional organization (e.g., establishing shared attention). We discuss these papers in our section on *Interactional Organization of Remote Healthcare*. These studies suggest that whereas telephone consultations are, at least to some extent, routinized, video consultations are still a novel interactional environment, used in a broad range of different clinical settings, with participants finding their way in developing some recognizable organization.

Consider the study on telephone health helplines by Lopriore et al. (2017). In line with other health helplines (Bloch & Leydon, 2019), opening sequences in these calls are streamlined, showing the nature of the call as providing a service. They are initiated by the call-taking nurse who answers the phone on behalf of the health helpline using a practice that is characteristic of a service call (e.g., “Healthdirect Australia, this is Karl, how can I help”).

Researchers find, however, that openings of video consultations are diverse. Pappas and Seale (2009) found that in cardiology and vascular surgery consultations, the novelty of the setting can have a “destabilising effect” on the organization of the talk, in which participants need to (re-)establish the relevant interactional frame. However, their study took place at a time when video was a more unfamiliar medium of care. More recent work by Stommel et al. (2019) and Ilomäki and Ruusuvuori (2020) indicates that while participants are still finding their way with the new technology, consistent practices are beginning to emerge. Both show that clinicians in different settings in different countries use a how-are-you type question following the greeting exchange, to not only elicit a problem presentation or other care-relevant information, but also establish that the connection is working.

Following the opening, patients are engaged in problem presentation. Three studies address this activity in telehealth (Hewitt et al., 2010; Lopriore et al., 2017; Stommel & Van Der Houwen, 2015), all indicating that the different media are used for specific types of clinical encounters. Hewitt et al. (2010) and Lopriore et al. (2017) showed that in telephone consultations patients present fewer concerns and clinicians asked fewer questions than they would for in-person encounters. Participants thereby treat the telephone consultation as more of a triage encounter. In a study on text-based counseling, Stommel and Van Der Houwen (2015) compared e-mail with chat and found that in e-mail, counselors create room for patients to present a range of issues, whereas in chat they provide limited space. Here too, we see an orientation to the affordances of the medium. E-mail is asynchronous and offers participants a single turn of undefined length, where the counselor cannot “interrupt.” Chat is more synchronous, providing for shorter turns, and thus faster opportunities for counselors to come in.

One study investigates a distinct form of healthcare where parents submit videos of their child to the clinician and where they discuss these videos with a pediatric occupational therapist over text (Dalmaijer et al., 2023). This allows parents to show not only how their child is progressing, but also how they implement the clinician’s advice. Pediatricians in turn can provide targeted advice and feedback by referring to specific time stamps in the videos and describing the exact behavior that they saw and which they considered relevant.

Affordances of each technology shape the possibilities for healthcare activities. Much of the literature on telehealth focuses on the limitations and challenges of remote modalities (e.g., the lack of touch for physical examinations). CA studies demonstrate that and show how participants accomplish such activities and use remote media for specific clinical and interactional goals that are tailored to the affordances of the medium. Both the participants and the researchers demonstrate that remote healthcare encounters are not defective forms of in-person healthcare, but different forms of care.

## Applications

Evidence-based guidance and resources on communication in telehealth are greatly needed (Shaw et al., 2020). While telephone has been part of the medical practice of services for a long time, the scale-up of telehealth has been so rapid that, at the time of writing, most clinicians have received no or limited training in how to conduct consultations by telephone, text or video. Much of the evidence base for remote clinical communication is based on small scale studies and post-hoc methods like surveys and interviews that only capture recollected experiences (Seuren & Shaw, 2022). Many studies of interaction use quantitative coding approaches, limiting their capacity to capture the richness of clinical encounters, especially as they are rarely adapted to technology (Ford & Reuber, 2023; Seuren & Shaw, 2022) Recommendations are generic and abstract, telling clinicians to pay attention to such things as rapport, building a therapeutic relationship, and attending to non-verbal communication and eye-contact (Connolly et al., 2020). However, it is unclear that these are actual problems in remote consultations. These recommendations also offer no insights into how clinicians could go about addressing them – for example, what are the practices by which one builds a therapeutic relationship in text, telephone or video? Through rigorous analysis of empirical data, CA is uniquely positioned to deliver evidence-based recommendations for remote clinical practice.

Of the 41 papers considered in this review, 20 discussed how to improve clinical practice. Their applications took one of two forms. Most papers (n = 17) recommend changes to clinical practice, either through specific interactional practice(s) that clinicians could implement (e.g., ask patients if they have additional concerns (White et al., 2022)) or through training programs (Pappas & Seale, 2009, 2010). These recommendations are based on observations of practices that worked or did not work well, and extrapolations from existing CA literature.

**Figure 2.**
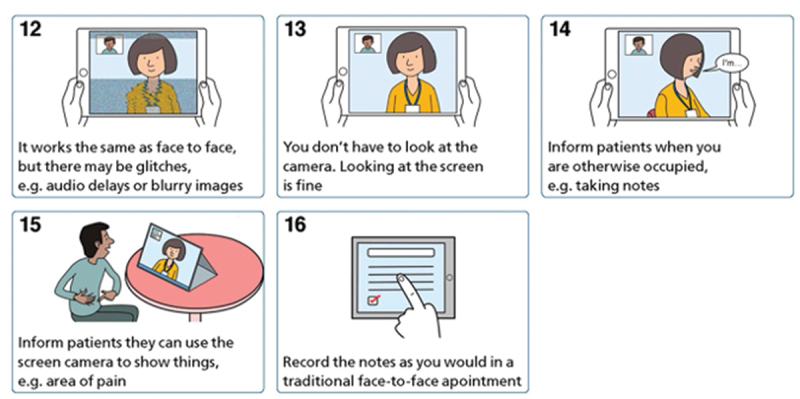
Extract from CA-informed resources on video consulting for clinicians.

The second application is for researchers to develop workshops and/or practical resources (e.g., illustrated or animated guidance) that address the complete encounter. While workshops have become more established forms of disseminating CA findings (Stokoe, 2014), practical resources are novel. The benefit of a workshop is that it can be tailored to the specific clinical context, allowing for direct engagement with practitioners, and can serve as a motivation for organizations to work with CA researchers. Stommel and te Molder (2015a) developed a workshop for the specific counseling organization where they collected the data, as a way to give back to the organization. Resources are more generic, can incorporate findings from mixed-methods studies and are designed to be used by diverse groups of stakeholders. Shaw et al. (2020) developed illustrated and animated resources, informed by the findings from their research as well as the broader literature, and co-designed through series of workshops with patients and clinicians with lived experience (see Figure 2).

With all these approaches to dissemination, success is conditional on implementation. Yet in most studies, the practical applications are an afterthought, typically presented as a single sentence somewhere in the Discussion section. Only one study in our review had tested their recommendation to evaluate effectiveness (Stommel & te Molder, 2015b). And while Shaw et al. (2020) worked with NHS England to develop, brand and disseminate resources, evidence on uptake and impact has not been collected.

For CA to shape the narrative around telehealth, it is crucial that we not only make recommendations, but demonstrate that they work and that we actively develop strategies for successful implementation of these recommendations across healthcare services.

It is up to funding agencies to facilitate this process. While the value of qualitative methods in health services research is slowly getting recognition with national health research funders like NIHR and NIH, micro-analytic research of interaction in health encounters is still underappreciated. The proven track record of CA in health encounters and mediated interaction would and should make it the gold-standard approach for investigating and supporting implementation, spread and scale-up of telehealth. For similar arguments in a different area of healthcare, see Riou (2024). For further comments on funding, see Parry and Barnes (2024).

## Issues for reflection

### Telehealth as a single focus of study

Our focus in this review has been on studies of telehealth that investigate how the affordances of the medium affect the interaction. However, there are two critical methodological issues. First, as per our Methods section, we excluded studies that investigate interaction in its own right. These did not analyze whether, and if so how, the mediated nature of the encounter mattered for the participants. From a CA perspective, our focus is potentially controversial. We cannot a priori distinguish between the interaction and its medium. The medium is just another part of the context (Raclaw, 2009), in the same way as any part of the setting of the encounter (including its institutional nature). Its relevance for the interaction should be an analytical finding in its own right. In other words, those researching telehealth need not justify the relevance of their study based on a presumed difference between the interaction in the remote medium and in-person care.

We chose our approach as we were interested in studies that analyzed telehealth as telehealth, and not simply as clinical interaction. For certain audiences, such as healthcare professionals and policymakers, the differences between telehealth and in-person care are a central concern. Their questions are when telehealth is safe, appropriate, and what can be accomplished in a remote consultation. Particularly in these cases, CA research can investigate the interaction as mediated interaction: how the affordances of the medium of the encounter matter (or do not matter) for the participants. The medium need not be presumed to be deficient.

The second issue is our use of the label “telehealth,” which is not a uniform category. Video, telephone, and the various forms of text all have their own affordances. The only thing they have in common is that they lack a shared physical space. Not even (a)synchronicity is a distinguishing feature. As Dalmaijer et al. (2023) show, healthcare can involve multiple modalities interchangeably, videos submitted by patients and text in response by paramedics. Clinical disciplines like dermatology are using photos or videos submitted asynchronously by patients in combination with synchronous video or telephone (Wang et al., 2022). Asynchronous video has also been documented in non-institutional interaction as its own unique form of social interaction (Rintel et al., 2016). Precisely because all these media are distinct, we took them as our combined analytical focus. While research on video is currently more prevalent, telephone is by far the dominant remote option in primary care (Greenhalgh et al., 2022), and there is an extensive body of CA research on text-based care. Our aim was to provide a state-of-the-art review on telehealth, and we saw no reason to prioritize one medium over another.

### Quality of evidence

CA has a long tradition investigating mediated interaction, however research on telehealth is still relatively recent. We found no publications from before 2005 and most studies on video consultations have been published since 2020. Researchers thus have little experience with investigating telehealth. These matters raise concerns about research quality and the extent to which study findings are transferable.

Parry and Land (2013) propose that when reviewing CA research, there are two criteria for quality: analytic breadth, how widely a practice is studied (e.g., amount of data, number of settings), and “the detail and depth of analysis” (e.g., interactional resources, sequential implications). Many phenomena in telehealth have received sparse attention, as papers are generally limited to one clinical setting and sometimes only a few participants. The state-of-the-art in this field thus provides relatively weak evidence for the transferability and clinical applicability of these findings. However, while many have small data sets, sometimes even single cases (e.g., Due, 2021; Due & Lange, 2020), these studies are characterized and even strengthened by their singular analytic depth: they offer rigorous analyses of how participants manage technology and thus what the affordances of telehealth are for them.

Studies in this review also point to a new criterion for quality: the adequacy of the collected data. The fractured ecologies of telehealth, especially text and video, provide additional challenges for researchers collecting data. On the one hand, the goal is to provide a participants’ perspective, which means that researchers should rely only on the materials that participants can use (Olbertz-Siitonen, 2015). On the other hand, using video recordings from both sides of the video calls enables appreciation of what each participant sees and does, and the extent of fractured ecologies (Seuren et al., 2021).

**Figure 3.**
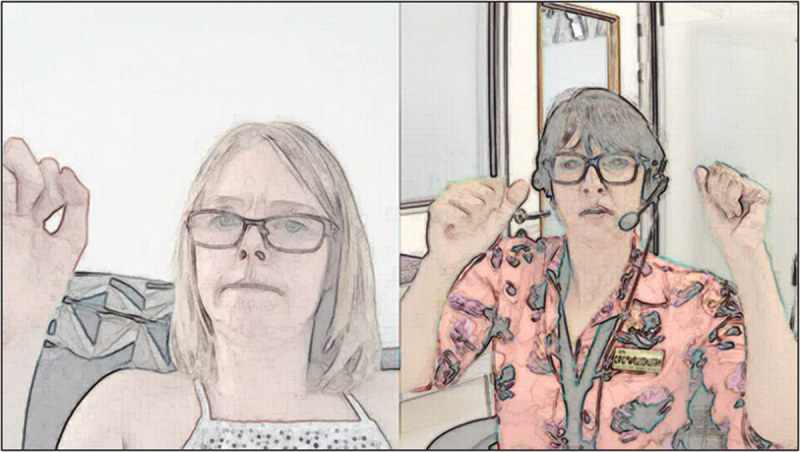
Screenshot of video consultation recorded in MS teams with the patient’s arms not fully visible during assessment.

For text-based consultations, particularly chat, researchers can rely on e-mail or chat logs, but these logs do not capture how each post was constructed by the participants, and what they may be doing other than typing (Meredith & Stokoe, 2014). For video consultations, studies have used data collection approaches as extensive as combining cameras in both the clinic and patient’s home with screen-capture software, resulting in four parallel recordings (Seuren et al., 2020), to recording one side of the call with a single camera (Stommel et al., 2019). Where healthcare services use videoconferencing software such as MS Teams, these can be used to record the encounter (Seuren & Shaw, 2024). However, these recordings offer a split-screen view, meaning only a limited view of each participant and their local physical ecology of action (see Figure 3).

Researchers have to weigh “completeness” of the data against the feasibility and practicality of collecting them, as well as the imposition data collection makes on the research participants.^3^^3^This is partially a consideration for Review Ethics Committees, who have limited to no experience with qualitative studies of remote interaction, but researchers will also need to make their own assessments. More extensive data allow for broader analyses (e.g., examining the non-mutual realities), but limited data can be just as good for more targeted questions (e.g., practices for doing other attentiveness).

## The future of CA and telehealth

Telehealth will continue to be a large part of healthcare services. As we have shown, research on the various technologies remains limited. This dearth of knowledge on telehealth offers a great opportunity for CA scholars to provide insights into how remote healthcare encounters are accomplished, what kind of communication practices work well, which ones do not, and how we can enable patients, clinicians and support staff to optimally use these new service models (Seuren & Shaw, 2022).

CA research on telehealth has recently focused almost exclusively on video consulting. While this form of telehealth has become more prevalent, it is less ubiquitous than telephone consulting, which is increasingly common, especially in primary care, but highly under-researched. As Barnes and Woods (2024) argue, in the context of increasing use of telephone consultations, these should be on the agenda for future CA research. There are other domains CA should focus on. Healthcare services are increasingly hybrid, with patients seeing their clinician in-person for specific types of encounters (e.g., acute care) and remotely for others (e.g., follow-up). Furthermore, polymedia service models in which patients, for example, submit e-consultations (text), photos or videos to their healthcare provider, which they then discuss in the consultation or through text exchange (as, for example, in Dalmaijer et al., 2023) are increasingly common. For CA to be relevant for clinical practice, we need to conduct research on these more prevalent forms of mediated interaction and on emerging forms of hybrid and polymedia care.

With remote media being increasingly integrated into healthcare services, investigating the complexity of interactions across technological media offers an exciting new area for CA research, informing not only our understanding of healthcare services, but social interaction in an increasingly digital world.

## Supplementary Material

Supplemental Material

## References

[cit0001] Arminen, I., Licoppe, C., & Spagnolli, A. (2016). Respecifying mediated interaction. *Research on Language and Social Interaction*, 49(4), 290–309. 10.1080/08351813.2016.1234614

[cit0002] Australia Digital Health Agency. (2018). *Safe, seamless and secure: Evolving health and care to meet the needs of modern Australia*. Government of Australia.

[cit0003] Barnes, R., & Woods, C. (2024). Communication in primary healthcare: A state-of-the-art literature review of conversation-analytic research. *Research on Language and Social Interaction*, 57(1), 7–37. 38707494 10.1080/08351813.2024.2305038 PMC11067862

[cit0004] BC Ministry of Health. (2015). Primary and community care in BC: A strategic policy framework. *Cross sector policy discussion paper*.

[cit0005] Bloch, S., & Leydon, G. (2019). Conversation analysis and telephone helplines for health and illness: A narrative review. *Research on Language and Social Interaction*, 52(3), 193–211. 10.1080/08351813.2019.1631035

[cit0006] Buchholz, M. B., & Kachele, H. (2015). Emergency SMS-based intervention in chronic suicidality: A research project using conversation analysis. In J. S. Scharff (Ed.), *Psychoanalysis online 2: Impact of technology on development, training, and therapy* (pp. 145–162). Karnac Books.

[cit0007] Byrne, P., & Long, B. (1976). *Doctors talking to patients: A study of the verbal behaviour of general practitioners consulting in their surgeries*. Her Majesty’s Stationary Office.

[cit0008] Chang, J. E., Lai, A. Y., Gupta, A., Nguyen, A. M., Berry, C. A., & Shelley, D. R. (2021). Rapid transition to telehealth and the digital divide: Implications for primary care access and equity in a post-COVID era. *The Milbank Quarterly*, 99(2), 340–368. 10.1111/1468-0009.12509 34075622 PMC8209855

[cit0009] Chatwin, J., Bee, P., Macfarlane, G. J., & Lovell, K. (2014). Observations on silence in telephone delivered cognitive behavioural therapy (T-CBT). *Journal of Applied Linguistics and Professional Practice*, 11(1), 1–22. 10.1558/japl.27652

[cit0010] Cipolletta, S., Frassoni, E., & Faccio, E. (2018). Construing a therapeutic relationship online: An analysis of videoconference sessions. *Clinical Psychologist*, 22(2), 220–229. 10.1111/cp.12117

[cit0011] Clarke, G. M., Dias, A., & Wolters, A. (2022). *Access to and delivery of general practice services: A study of patients at practices using digital and online tools*. The Health Foundation.

[cit0012] Connolly, S. L., Miller, C. J., Lindsay, J. A., & Bauer, M. S. (2020). A systematic review of providers’ attitudes toward telemental health via videoconferencing [Review]. *Clinical Psychology: Science and Practice*, 27(2), Article e12311. 10.1111/cpsp.12311 PMC9367168 35966216

[cit0013] Dalley, D., Rahman, R., & Ivaldi, A. (2020). Health care professionals’ and patients’ management of the interactional practices in telemedicine videoconferencing: A conversation analytic and discursive systematic review. *Qualitative Health Research*, 31(4), 804–814. 10.1177/1049732320942346 32741261

[cit0014] Dalmaijer, E., Pas, B., Spooren, W., & Stommel, W. (2023). How technology shapes advice: Professional-parent interaction in digital pediatric treatment. *Frontiers of Communication*, 8. 10.3389/fcomm.2023.1205883

[cit0015] Drew, P. (1998). “Out-of-Hours” calls to the doctor: Misalignment between callers and doctor during diagnostic questioning. In S. Cmejrkova, J. Hoffmannova, O. Mullerova, & J. Svetla (Eds.), *Dialoganalyse* (Vol. I/2, pp. 65–78). De Gruyter. 10.1515/9783110965049-007

[cit0016] Drew, P. (2006). Misalignments in “after-hours” calls to a British GP’s practice: A study in telephone medicine. In J. Heritage & D. W. Maynard (Eds.), *Communication in medical care: Interaction between primary care physicians and patients* (pp. 416–444). Cambridge University Press.

[cit0017] Due, B. L. (2021). RoboDoc: Semiotic resources for achieving face-to-screenface formation with a telepresence robot. *Semiotica*, 2021(238), 253–278. 10.1515/sem-2018-0148

[cit0018] Due, B. L. (2022). Situated co-operative creativity. *Pragmatics and Society*, 13(4), 684–702. 10.1075/ps.20031.due

[cit0019] Due, B. L., & Lange, S. B. (2020). Body part highlighting: Exploring two types of embodied practices in two sub-types of showing sequences in video-mediated consultations. *Social Interaction. Video-Based Studies of Human Sociality*, 3(3). 10.7146/si.v3i3.122250

[cit0020] Ekberg, S., Barnes, R., Kessler, D., Malpass, A., & Shaw, A. (2013). Managing the therapeutic relationship in online cognitive behavioural therapy for depression: Therapists’ treatment of clients’ contributions. *Language@Internet*, 10, 1–18. https://www.languageatinternet.org/articles/2013/Ekberg

[cit0021] Ekberg, S., Barnes, R., Kessler, D., Malpass, A., & Shaw, A. (2016). Managing clients’ expectations at the outset of online Cognitive Behavioural Therapy (CBT) for depression. *Health Expectations*, 19(3), 557–569. 10.1111/hex.12227 25088009 PMC5055246

[cit0022] Ekberg, S., Danby, S., Theobald, M., Fisher, B., & Wyeth, P. (2019). Using physical objects with young children in ‘face-to-face’ and telehealth speech and language therapy. *Disability & Rehabilitation*, 41(14), 1664–1675. 10.1080/09638288.2018.1448464 29566569

[cit0023] Ekberg, S., Shaw, A. A., Kessler, D., Malpass, A., & Barnes, R. (2016). Orienting to emotion in computer-mediated cognitive behavioral therapy. *Research on Language and Social Interaction*, 49(4), 310–324. 10.1080/08351813.2016.1199085

[cit0024] Ford, J., & Reuber, M. (2023). Comparisons of communication in medical face-to-face and teleconsultations: A systematic review and narrative synthesis. *Health Commun*, 1–15. 10.1080/10410236.2023.2201733 37092952

[cit0025] Gibson, J. J. (1979). *The ecological approach to visual perception*. Psychology Press. 10.4324/9781315740218

[cit0026] Goodwin, C. (1994). Professional vision. *American Anthropologist*, 96(3), 606–633. 10.1525/aa.1994.96.3.02a00100

[cit0027] Greenhalgh, T., Ladds, E., Hughes, G., Moore, L., Wherton, J., Shaw, S. E., Papoutsi, C., Wieringa, S., Rosen, R., Rushforth, A., & Rybczynska-Bunt, S. (2022). Why do GPs rarely do video consultations? Qualitative study in UK general practice. *British Journal of General Practice*, 72(718), e351–e360. 10.3399/BJGP.2021.0658 PMC8936181 35256385

[cit0028] Greenhalgh, T., Procter, R., Wherton, J., Sugarhood, P., & Shaw, S. (2012). The organising vision for telehealth and telecare: Discourse analysis. *BMJ Open*, 2(4), e001574. 10.1136/bmjopen-2012-001574 PMC3401833 22815469

[cit0029] Greenhalgh, T., Shaw, S., Wherton, J., Vijayaraghavan, S., Morris, J., Bhattacharya, S., Hanson, P., Campbell-Richards, D., Ramoutar, S., Collard, A., & Hodkinson, I. (2018). Real-World implementation of video outpatient consultations at macro, meso, and micro levels: Mixed-method study. *Journal of Medical Internet Research*, 20(4), e150. 10.2196/jmir.9897 29625956 PMC5930173

[cit0030] Greenhalgh, T., Wherton, J., Shaw, S., Papoutsi, C., Vijayaraghavan, S., & Stones, R. (2019). Infrastructure revisited: An ethnographic case study of how health information infrastructure shapes and constrains technological innovation. *Journal of Medical Internet Research*, 21(12), e16093. 10.2196/16093 31855184 PMC6940857

[cit0031] Hall Dykgraaf, S., Desborough, J., de Toca, L., Davis, S., Roberts, L., Munindradasa, A., McMillan, A., Kelly, P., & Kidd, M. (2021). “A decade’s worth of work in a matter of days”: The journey to telehealth for the whole population in Australia. *International Journal of Medical Informatics*, 151, 104483. 10.1016/j.ijmedinf.2021.104483 33984625 PMC8103781

[cit0032] Hansen, J. P. B. (2020). Invisible participants in a visual ecology: Visual space as a resource for organising video-mediated interpreting in hospital encounters. *Social Interaction. Video-Based Studies of Human Sociality*, 3(3). 10.7146/si.v3i3.122609

[cit0033] Heagarty, M. C. (1978). From house calls to telephone calls. *American Journal of Public Health*, 68(1), 14–15. 10.2105/ajph.68.1.14 623356 PMC1653837

[cit0034] Heath, C. (1981). The opening sequence in doctor-patient interaction. In P. Atkinson & C. Heath (Eds.), *Medical work: Realities and routines* (pp. 71–90). Gower.

[cit0035] Heath, C. (2006). Body work: The collaborative production of the clinical object. In J. Heritage & D. W. Maynard (Eds.), *Communication in medical care: Interaction between primary care physicians and patients* (pp. 185–213). Cambridge University Press.

[cit0036] Heritage, J., & Robinson, J. D. (2006). The structure of patients’ presenting concerns: Physicians’ opening questions. *Health Communication*, 19(2), 89–102. 10.1207/s15327027hc1902_1 16548700

[cit0037] Hewitt, H., Gafaranga, J., & McKinstry, B. (2010). Comparison of face-to-face and telephone consultations in primary care: Qualitative analysis. *British Journal of General Practice*, 60(574), e201–e212. 10.3399/bjgp10X501831 PMC2858552 20423575

[cit0038] Hutchby, I. (2001a). *Conversation and technology: From the telephone to the internet*. Polity Press.

[cit0039] Hutchby, I. (2001b). Technologies, texts and affordances. *Sociology*, 35(2), 441–456. 10.1177/S0038038501000219

[cit0040] Ilomaki, S., & Ruusuvuori, J. (2022). Preserving client autonomy when guiding medicine taking in telehomecare: A conversation analytic case study. *Nurs Ethics*, 9697330211051004. 10.1177/09697330211051004 PMC9127937 35119321

[cit0041] Ilomäki, S., & Ruusuvuori, J. (2020). From appearings to disengagements: Openings and closings in video-mediated tele-homecare encounters. *Social Interaction. Video-Based Studies of Human Sociality*, 3(3). 10.7146/si.v3i3.122711

[cit0042] Ilomaki, S., Ruusuvuori, J., & Laitinen, J. (2021). Effects of transmission delay on client participation in video-mediated group health counseling. *Qualitative Health Research*, 31(12), 2328–2339. 10.1177/10497323211010726 34014131 PMC8564242

[cit0043] Jager, M., & Stommel, W. (2017). The risk of metacommunication to manage interactional trouble in online chat counseling. *Linguistik Online*, 87(8), 191. 10.13092/lo.87.4179

[cit0044] Kevoe-Feldman, H. (2019). Inside the emergency service call-center: Reviewing thirty years of language and social interaction research. *Research on Language and Social Interaction*, 52(3), 227–240. 10.1080/08351813.2019.1631038

[cit0045] Licoppe, C. (2017). Skype appearances, multiple greetings and ‘coucou’: The sequential organization of video-mediated conversation openings. *Pragmatics*, 27(3), 351–386. 10.1075/prag.27.3.03lic

[cit0046] Licoppe, C., Luff, P. K., Heath, C., Kuzuoka, H., Yamashita, N., & Tuncer, S. (2017). *Showing objects: Holding and manipulating artefacts in video-mediated collaborative settings* 2017. CHI Conference on Human Factors in Computing Systems. 10.1145/3025453.3025848

[cit0047] Licoppe, C., & Morel, J. (2012). Video-in-interaction: “Talking Heads” and the multimodal organization of mobile and skype video calls. *Research on Language & Social Interaction*, 45(4), 399–429. 10.1080/08351813.2012.724996

[cit0048] Lopriore, S., LeCouteur, A., Ekberg, K., & Ekberg, S. (2019). “You’ll have to be my eyes and ears”: A conversation analytic study of physical examination on a health helpline. *Journal of Clinical Nursing*, 28(1–2), 330–339. 10.1111/jocn.14638 30091493

[cit0049] Lopriore, S., LeCouteur, A., Ekberg, S., & Ekberg, K. (2017). Delivering healthcare at a distance: Exploring the organisation of calls to a health helpline. *International Journal of Medical Informatics*, 104, 45–55. 10.1016/j.ijmedinf.2017.05.001 28599816

[cit0050] Luff, P. K., Heath, C., Kuzuoka, H., Hindmarsh, J., Yamazaki, K., & Oyama, S. (2003). Fractured ecologies: Creating environments for collaboration. *Human–Computer Interaction*, 18(1–2), 51–84. 10.1207/S15327051HCI1812_3

[cit0051] Madianou, M., & Miller, D. (2012). *Migration and new media: Transnational families and polymedia*. Routledge.

[cit0052] Meredith, J. (2019). Conversation analysis and online interaction. *Research on Language and Social Interaction*, 52(3), 214–256. 10.1080/08351813.2019.1631040

[cit0053] Meredith, J., & Stokoe, E. (2014). Repair: Comparing Facebook ‘chat’ with spoken interaction. *Discourse & Communication*, 8(2), 181–207. 10.1177/1750481313510815

[cit0054] Mlynář, J., González-Martínez, E., & Lalanne, D. (2018). Situated organization of video-mediated interaction: A review of ethnomethodological and conversation analytic studies. *Interacting with Computers*, 30(2), 73–84. 10.1093/iwc/iwx019

[cit0055] NHS. (2019). The NHS long term plan. NHS.

[cit0056] Nielsen, A. M. R. (2020). Co-constructing the video consultation-competent patient. *Social Interaction. Video-Based Studies of Human Sociality*, 3(3). 10.7146/si.v3i3.122708

[cit0057] Ohannessian, R., Duong, T. A., & Odone, A. (2020). Global telemedicine implementation and integration within health systems to fight the COVID-19 pandemic: A call to action. *JMIR Public Health and Surveillance*, 6(2), e18810. 10.2196/18810 32238336 PMC7124951

[cit0058] Olbertz-Siitonen, M. (2015). Transmission delay in technology-mediated interaction at work. *PsychNology Journal*, 13(2–3), 203–234.

[cit0059] Ontario Ministry of Health. (2019). Digital first for health. Ontario Ministry of Health. Retrieved September 27, from https://news.ontario.ca/en/release/54594/ontario-expanding-digital-and-virtual-health-care

[cit0060] Pappas, Y., & Seale, C. (2009). The opening phase of telemedicine consultations: An analysis of interaction. *Social Science & Medicine*, 68(7), 1229–1237. 10.1016/j.socscimed.2009.01.011 19201514

[cit0061] Pappas, Y., & Seale, C. (2010). The physical examination in telecardiology and televascular consultations: A study using conversation analysis. *Patient Education and Counseling*, 81(1), 113–118. 10.1016/j.pec.2010.01.005 20144523

[cit0062] Parry, R. H., & Barnes, R. K. (2024). Conversation-analytic research on communication in healthcare: Growth, gaps and potential. *Research on Language and Social Interaction*, 57(1), 1–6. 10.1080/08351813.2024.2305038 PMC11067862 38707494

[cit0063] Parry, R. H., & Land, V. (2013). Systematically reviewing and synthesizing evidence from conversation analytic and related discursive research to inform healthcare communication practice and policy: An illustrated guide. *BMC Medical Research Methodology*, 13(1). 10.1186/1471-2288-13-69 PMC3674894 23721181

[cit0064] Raclaw, J. (2009). Approaches to “Context” within conversation analysis. *Colorado Research in Linguistics*, 22. 10.25810/qbrs-0970

[cit0065] Rintel, S., Harper, R., & O’Hara, K. (2016). The tyranny of the everyday in mobile video messaging. CHI Conference on Human Factors in Computing Systems, New York, NY. 10.1145/2858036.2858042

[cit0066] Riou, M. (2024). Communication in prehospital and emergency care: A state-of-the-art literature review of conversation-analytic research. *Research on Language and Social Interaction*, 57(1), 55–72. 10.1080/08351813.2024.2305045 PMC11090155 38741749

[cit0067] Robinson, J. D. (1998). Getting down to business talk, gaze, and body orientation during openings of doctor‐patient consultations. *Human Communication Research*, 25(1), 97–123. 10.1111/j.1468-2958.1998.tb00438.x

[cit0068] Robinson, J. D. (2003). An interactional structure of medical activities during acute visits and its implications for patients’ participation. *Health Communication*, 15(1), 27–59. 10.1207/S15327027HC1501_2 12553776

[cit0069] Ruhleder, K., & Jordan, B. (2001). Co-constructing non-mutual realities: Delay-generated trouble in distributed interaction [journal article]. *Computer Supported Cooperative Work (CSCW)*, 10(1), 113–138. 10.1023/a:1011243905593

[cit0070] Savenstedt, S., Zingmark, K., Hyden, L.-C., & Brulin, C. (2005). Establishing joint attention in remote talks with the elderly about health: A study of nurses’ conversation with elderly persons in teleconsultations. *Scandinavian Journal of Caring Sciences*, 19(4), 317–324. 10.1111/j.1471-6712.2005.00346.x 16324054

[cit0071] Schegloff, E. A. (1991). Reflections on talk and social structure. In D. Boden & D. H. Zimmerman (Eds.), *Talk and social structure: Studies in ethnomethodology and conversation analysis* (pp. 44–70). University of California Press.

[cit0072] Schönfeldt, J., & Golato, A. (2003). Repair in chats: A conversation analytic approach. *Research on Language & Social Interaction*, 36(3), 241–284. 10.1207/S15327973RLSI3603_02

[cit0073] Seuren, L. M., Gilbert, A., Ramdharry, G., Walumbe, J., & Shaw, S. E. (2024). Video analysis of communication by physiotherapists and patients in video consultations: A qualitative study using conversation analysis. *Physiotherapy*, 123, 30–37. 10.1016/j.physio.2023.10.002 38262264

[cit0074] Seuren, L. M., & Shaw, S. E. (2022). Using linguistic ethnography to study video consultations: A call to action and future research Agenda. *Qualitative Health Research*, 32(5), 800–813. 10.1177/10497323221077297 35245150 PMC9152594

[cit0075] Seuren, L. M., Wherton, J., Greenhalgh, T., Cameron, D., A’Court, C., & Shaw, S. E. (2020). Physical examinations via video for patients with heart failure: Qualitative study using conversation analysis. *Journal of Medical Internet Research*, 22(2), e16694. 10.2196/16694 32130133 PMC7059096

[cit0076] Seuren, L. M., Wherton, J., Greenhalgh, T., & Shaw, S. E. (2021). Whose turn is it anyway? Latency and the organization of turn-taking in video-mediated interaction. *Journal of Pragmatics*, 172, 63–78. 10.1016/j.pragma.2020.11.005 33519050 PMC7819463

[cit0077] Shaw, S. E., Hughes, G., Wherton, J., Moore, L., Rosen, R., Papoutsi, C., Rushforth, A., Morris, J., Wood, G. W., Faulkner, S., & Greenhalgh, T. (2021). Achieving spread, scale up and sustainability of video consulting services during the COVID-19 pandemic? Findings from a comparative case study of policy implementation in England, Wales, Scotland and Northern Ireland. *Frontiers in Digital Health*, 3, 754319. 10.3389/fdgth.2021.754319 34988546 PMC8720935

[cit0078] Shaw, S. E., Seuren, L. M., Wherton, J., Cameron, D., A’Court, C., Vijayaraghavan, S., Morris, J., Bhattacharya, S., & Greenhalgh, T. (2020). Video consultations between patients and clinicians in diabetes, cancer, and heart failure services: A linguistic ethnographic study of video-mediated interaction. *Journal of Medical Internet Research*, 22(5), e18378. 10.2196/18378 32391799 PMC7248806

[cit0079] Stokoe, E. (2014). The Conversation Analytic Role-play Method (CARM): A method for training communication skills as an alternative to simulated role-play. *Research on Language and Social Interaction*, 47(3), 255–265. 10.1080/08351813.2014.925663

[cit0080] Stommel, W. (2012). Salutations, closings and pronouns: Some aspects of recipient design in online counselling. *Communication and Medicine*, 9(2), 145–158. 10.1558/cam.v9i2.145 24498699

[cit0081] Stommel, W., Licoppe, C., & Stommel, M. (2020). “Difficult to assess in this manner”: An “ineffective” showing sequence in post-surgery video consultation. *Social Interaction: Video-Based Studies of Human Sociality*, 3(3). 10.7146/si.v3i3.122581

[cit0082] Stommel, W., & Stommel, M. (2021). Participation of companions in video-mediated medical consultations: A microanalysis. In J. Meredith, D. Giles, & W. Stommel (Eds.), *Analysing digital interaction* (pp. 177–203). Palgrave MacMillan. 10.1007/978-3-030-64922-7_9

[cit0083] Stommel, W., & te Molder, H. (2015a). Counseling online and over the phone: When preclosing questions fail as a closing device. *Research on Language and Social Interaction*, 48(3), 281–300. 10.1080/08351813.2015.1058605

[cit0084] Stommel, W., & te Molder, H. (2015b). When technological affordances meet interactional norms: The value of pre-screening in online chat counseling. *PsychNology Journal*, 13(2–3), 235–258.

[cit0085] Stommel, W., & van der Houwen, F. (2013). Formulations in ‘Trouble’ chat sessions. *Language@Internet*, 10, 1–19. https://www.languageatinternet.org/articles/2013/stommel

[cit0086] Stommel, W., & Van Der Houwen, F. (2015). Counseling and new media technologies: A comparison of problem presentations in e-mail and in chat. *Communication & Medicine*, 12(2–3), 243–256. 10.1558/cam.18298 29048865

[cit0087] Stommel, W., van Goor, H., & Stommel, M. (2019). Other-Attentiveness in video consultation openings: A conversation analysis of video-mediated versus face-to-face consultations. *Journal of Computer-Mediated Communication*, 24(6), 353. 10.1093/jcmc/zmz015

[cit0088] Stommel, W., van Goor, H., & Stommel, M. (2020). The impact of video-mediated communication on closed wound assessments in postoperative consultations: Conversation analytical study. *Journal of Medical Internet Research*, 22(5), e17791. 10.2196/17791 32310816 PMC7238083

[cit0089] van Dael, J., Gillespie, A., Neves, A. L., & Darzi, A. (2022). Patient-clinician communication research for 21st century health care. *British Journal of General Practice*, 72(715), 52–53. 10.3399/bjgp22X718277 PMC8813107 35091398

[cit0090] Wang, R. H., Barbieri, J. S., Kovarik, C. L., & Lipoff, J. B. (2022). Synchronous and asynchronous teledermatology: A narrative review of strengths and limitations. *Journal of Telemedicine and Telecare*, 28(7), 533–538. 10.1177/1357633X221074504 35108130

[cit0091] White, S. J., Nguyen, A., & Cartmill, J. A. (2022). Agency and the telephone: Patient contributions to the clinical and interactional agendas in telehealth consultations. *Patient Education and Counseling*, 105(7), 2074–2080. 10.1016/j.pec.2022.01.004 35074218 PMC9595389

[cit0092] Wong, M. Y. Z., Gunasekeran, D. V., Nusinovici, S., Sabanayagam, C., Yeo, K. K., Cheng, C. Y., & Tham, Y. C. (2021). Telehealth demand trends during the COVID-19 pandemic in the top 50 most affected countries: Infodemiological evaluation. *JMIR Public Health and Surveillance*, 7(2), e24445. 10.2196/24445 33605883 PMC7899203

[cit0093] World Health Organization. (2021). Implementing telemedicine services during COVID-19: Guiding principles and considerations for a stepwise approach (WPR/DSE/2020/032).

