# Supplementary material for: Communication in Telehealth: A State-of-the-Art Literature Review of Conversation-Analytic Research
Source: Res Lang Soc Interact. 2024 Apr 3;57(1):73–90. doi: 10.1080/08351813.2024.2305045 (PMC11090155; doi:10.1080/08351813.2024.2305045)
Supplement: Supplemental Material [file HRLS_A_2305045_SM5204.zip › Supplementary file A_2305045.pdf]

Supplementary File

Communication in Telehealth: A state-of-the-art review of conversation analytic research

Seuren et al *Rolsi* 2024

Search approach for state-of-the-art review on CA and Telehealth

We conducted a systematic search of PubMed, Scopus, LLBA, PsycLink, and Web of Science using combinations of methodological search terms on “Conversation Analysis”, “Discursive Psychology”, “Ethnomethodology, “Interactional Linguistics", “Discourse Analysis" and “Interaction Analysis", with topical search terms “telemedicine,” “telehealth”, “e-health”, “ehealth”, “out-of-hours”, “after-hours”, “e-consultation”, and combinations of “video”, “virtual”, “remote”, “digital”, “Skype, “facetime”, “telephone”, “virtual”, “email”, “e-mail”, “sms”, “whatsapp”, “asynchronous”, “computer-mediated” and “chat”, with “consultation”, “counselling”, “therapy” and “psychotherapy”.

After removing all duplicates, this search resulted in 965 unique publications. We initially included articles that (a) used CA as their main or one of their main research methods, (b) focused on naturally occurring interaction in telehealth, either (quasi-)synchronous or asynchronous, (c) analysed encounters between healthcare professionals and patients, (d) investigated how the remote nature was procedurally consequential for the interaction, (e) used transcripts of their data to support the analysis, (f) involved original, empirical work (e.g., we excluded protocols and reviews) and (g) had been peer-reviewed, including forthcoming, “online first,” publications. Publications had to be written in English but could rely on data in other languages. We excluded telephone helplines and emergency lines, as there have been recent reviews discussing the state of the art on these topics (Bloch & Leydon, 2019; Kevoe-Feldman, 2019 - full references contained in the main paper).

Two authors (LS and ED) independently reviewed the first 200 papers in the selection against the inclusion and exclusion criteria based on the title and abstract. The authors agreed

Information Classification: General

on 96% of cases, with Cohen's Kappa measure for intercoder reliability at $\kappa = 0.56$. Disagreements were resolved through discussion. Five papers were excluded after re-reviewing the abstract, as they did not actually meet the inclusion criteria. For three papers, the authors consulted the full manuscript, before including one and excluding the other two. Following this initial selection, all authors agreed on two additional inclusion criteria, papers that: (a) used conversation analysis or ethnomethodology as a main method of research; (b) included text-based helplines for counselling (e.g., alcohol, drugs) or mental health (e.g., anxiety, CBT) (chat, SMS, email). The lead author then reviewed the rest of the collection against these criteria. This resulted in a collection of 32 original articles.

To complement our systematic search, we searched journals that routinely publish CA research (e.g., Research on Language and Social Interaction, Journal of Pragmatics, Social Interaction: Video-Based Studies of Human Sociality) as well as the EMCA Wiki[1]. We then used snowballing to explore if there were additional papers that were not returned in our searches. We searched Google Scholar for articles that had cited the papers that were returned in our search and emailed key authors from the initial search to ask if they had published any additional work on remote care. This resulted in an additional 18 articles. The large volume of additional articles seems to be partially a result of some journals not being indexed.

Thirteen of the 50 studies explored telehealth but did not focus on how either the remote or mediated nature of the healthcare encounter was procedurally consequential. After discussion, we decided to exclude these studies and only on studies that investigated how mediation shapes the consultation. While these studies discussed remote healthcare encounters, we did not consider them to be about remote healthcare encounters. While we appreciate this can be somewhat of a subjective assessment, we do not consider it dissimilar from how studies on in-person interaction need not focus on the in-person nature of the interaction as in some way relevant for the interaction. Interactional organisation and practices can and often do transcend settings and contexts. Without a discussion on how the interaction was, or was not, shaped by the mediated nature, it is unclear to what extent findings might be context-bound. Our final collection consisted of 37 original articles.

[1] http://emcawiki.net/Main_Page

Information Classification: General

We redid our search in April 2023 to include any studies that had been published between submission of our original and revised manuscript. After removing duplicates, this resulted in 69 potential articles. Of these, six met our inclusion criteria and of those, two had already been included in our original search as pre-publications through snowball sampling. We added four articles to our corpus, bringing our final collection to 41 original articles.

Information Classification: General
