# Supplementary material for: Communication in Telehealth: A State-of-the-Art Literature Review of Conversation-Analytic Research
Source: Res Lang Soc Interact. 2024 Apr 3;57(1):73–90. doi: 10.1080/08351813.2024.2305045 (PMC11090155; doi:10.1080/08351813.2024.2305045)
Supplement: Supplemental Material [file HRLS_A_2305045_SM5204.zip › Supplementary file B_2305045.pdf]

Supplementary File 2

Literature included in our state-of-the-art review

Article	Country and Language	Setting	Medium	Patient Participants	Recordings in data	Method of recording
Buchholz et al. (2015)	Germany, German	Psychotherapy	SMS	1	1	Text
Chatwin et al. (2014)	UK, English	CBT	Telephone	9	210	Audio
Cipolletta et al. (2018)	Italy, Italian	Psychotherapy	Video	5	15	Video
Dalmaijer et al. (under review)	Netherlands, Dutch	Paediatric Rehabilitation	Email, Video	7	376 videos, 799 texts	Text, Video
Due (2021)	Denmark, Danish	Rehabilitation	Video	1	1	Video
Due (2022)	Denmark, Danish	Unspecified	Video	1	1	Video
Due et al. (2020)	Denmark, Danish	Physiotherapy	Video	1	1	Video
Ekberg et al. (2013)	UK, English	Psychotherapy	Chat	22	149	Text
Ekberg et al. (2016a)	UK, English	Psychotherapy	Chat	183	-	Text
Ekberg et al. (2016b)	UK, English	Psychotherapy	Chat	183	1279	Text
Ekberg et al. (2019)	Australia, English	Speech & Language Therapy	Video	4	11	Video
Hansen (2020)	Norway, Norwegian	Various	Video	7	7	Video
Hewitt et al. (2020)	UK, English	Primary Care	Telephone	65	65	Audio
Ilomäki et al. (2020)	Finland, Finnish	Home care	Video	4	14	Video
Ilomäki et al. (2021)	Finland, Finnish	Diabetes Counselling	Video	3	19	Video
Ilomäki et al. (2022)	Finland, Finnish	Home care	Video	1	2	Video
Jager et al. (2017)	Netherlands, Dutch	Alcohol and Drugs Counselling	Chat	106	106	Text
Lopriore et al. (2017)	Australia, English	Health Helpline	Telephone	196	196	Audio
Lopriore et al. (2019)	Australia, English	Health Helpline	Telephone	196	196	Audio
Nielsen (2020)	Denmark, Danish	Care Home	Video	1	4	Video
Pappas et al. (2009)	UK, English	Cardiology	Video	10	10	Video
Pappas et al. (2010)	UK, English	Cardiology	Video	10	10	Video

Pappas et al. (2019)	UK, English	Cardiology	Video	10	10	Video
Savenstedt et al. (2005)	Sweden, Swedish	Care Home	Video	11	22	Video
Seuren et al. (2020)	UK, English	Heart Failure	Video	7	7	Video
Seuren et al. (2021)	UK, English	Heart Failure, Diabetes, Cancer	Video	25	25	Video
Seuren et al. (2022)	UK, English	Heart Failure, Diabetes, Cancer	Video	37	37	Video
Seuren & Shaw (under review)	UK, English	Heart Failure, Diabetes, Cancer, Physiotherapy	Video	52	52	Video
Shaw et al. (2020)	UK, English	Heart Failure, Diabetes, Cancer	Video	37	37	Video
Stommel (2012)	Netherlands, Dutch	Mental Health Counselling	Email	34	34	Text
Stommel et al. (2013)	Netherlands, Dutch	Mental Health Counselling	Chat	15	53	Text
Stommel et al. (2014)	Netherlands, Dutch	Mental Health Counselling	Email	20	20	Text
Stommel et al. (2015a)	Netherlands, Dutch	Alcohol and Drugs Counselling	Chat, telephone	80	80	Audio, Text
Stommel et al. (2015b)	Netherlands, Dutch	Alcohol and Drugs Counselling	Chat	74	74	Text
Stommel et al. (2015c)	Netherlands, Dutch	Mental Health Counselling	Chat, Email	37	37	Text
Stommel (2016)	Netherlands, Dutch	Alcohol and Drugs Counselling	Chat	49	49	Text
Stommel et al. (2019)	Netherlands, Dutch	Surgery	Video	39	39	Video
Stommel et al. (2020a)	Netherlands, Dutch	Surgery	Video	39	39	Video
Stommel et al. (2020b)	Netherlands, Dutch	Surgery	Video	39	39	Video
Stommel et al. (2021)	Netherlands, Dutch	Surgery	Video	22	22	Video
White et al. (2022)	Australia, English	Gastrointestinal	Telephone	15	14	Audio
